# The Relationship Between NETosis and Biofilm Formation in Chronic Infections

**DOI:** 10.3390/biom15121692

**Published:** 2025-12-04

**Authors:** Wafa Aziz, Hina Sultana, Vinay Kumar, Anuradha Tyagi

**Affiliations:** 1 Department of Medicine, Pennsylvania State University Hershey Medical Centre, Hershey, PA 17033, USA; wqa5122@psu.edu; 2 Integrative Program for Biological and Genome Sciences, University of North Carolina at Chapel Hill, Chapel Hill, NC 27599, USA; sultanah@email.unc.edu; 3 Department of cBRN, Institute of Nuclear Medicine & Allied Sciences, Delhi 110054, India

**Keywords:** biofilms, chronic infections, immune response, neutrophil extracellular traps (NETs), NETosis

## Abstract

Chronic infections pose significant clinical challenges due to their persistent nature, heightened resistance to conventional therapies, and association with biofilm formation. Neutrophil extracellular traps (NETs), released through a unique form of cell death known as NETosis, serve as an innate immune defense mechanism by trapping and neutralizing pathogens. However, accumulating evidence reveals a complex and paradoxical relationship between NETs and microbial biofilms. While NETs can immobilize and kill planktonic microbes, the extracellular DNA and associated proteins often contribute to biofilm stability, immune evasion, and chronic infection persistence. This review explores the bidirectional interactions between NETosis and biofilm formation, with a focus on their synergistic roles in the pathogenesis of chronic infections such as cystic fibrosis lung disease, diabetic foot ulcers, periodontitis, and implant-associated infections. We outline the molecular mechanisms governing NETosis, the structural and functional dynamics of biofilms, and how these processes intersect to form recalcitrant infection niches. Emerging therapeutic strategies aimed at disrupting this pathogenic interplay including DNase-based treatments, PAD4 inhibitors, and combination therapies are critically evaluated. By illuminating the pathogenic synergy between NETs and biofilms, this review underscores the need for integrated immunomodulatory and anti-biofilm interventions to effectively manage chronic infectious diseases and improve patient outcomes.

## 1. Introduction

Chronic infections pose serious challenges to global healthcare systems due to their prolonged course and the complex interplay of microbial persistence and host responses. These infections are associated with considerable clinical consequences, including increased morbidity, reduced quality of life, and elevated healthcare costs. Chronic respiratory infections, particularly those involving *Pseudomonas aeruginosa*, have been shown to impair lung function and decrease survival rates, notably in patients with cystic fibrosis [1].

Two key processes sustaining chronic infections are biofilm formation and NETosis. Biofilms, composed of bacterial communities embedded within a self-produced extracellular polymeric matrix (EPS), significantly increase resistance to antibiotics and immune clearance compared to planktonic cells [2]. Conversely, NETosis serves as a frontline immune defense, entrapping microbes in DNA-protein webs rich in antimicrobial factors [3]. Paradoxically, while neutrophil extracellular traps (NETs) are designed to restrict infection, their components, particularly extracellular DNA (eDNA) and histone-bound proteins, often integrate into the biofilm matrix, enhancing microbial stability, immune evasion, and antibiotic resistance [4]. Thus, the very mechanism intended to eliminate pathogens can inadvertently promote the persistence of infection.

Understanding the interaction between these processes holds potential for identifying new therapeutic strategies. By clarifying their mechanistic and pathological intersections, researchers can identify strategies to dismantle biofilms or modulate aberrant NET formation, ultimately improving the management of chronic, treatment-refractory infections. This review synthesizes current evidence on the molecular, immunological, and clinical dimensions of the NET–biofilm interaction, emphasizing its role in infection persistence and emerging opportunities for targeted intervention.

## 2. Biofilm Biology and Clinical Relevance

Biofilms are complex assemblies of microorganisms that grow attached to surfaces, encased in a protective layer of extracellular substances they secrete [5]. Their formation is a coordinated, multi-phase process beginning with reversible adhesion of planktonic cells via physicochemical forces, such as van der Waals interactions, electrostatic attractions, and hydrophobic effects [6,7]. This is followed by irreversible attachment, mediated by adhesins, pili, and fimbriae, alongside the synthesis of EPS components, including polysaccharides, eDNA, and proteins. These early structures mature into microcolonies and well-organized biofilms, where the EPS matrix provides mechanical stability and protection against environmental and host immune factors. Ultimately, biofilms disseminate through erosion, sloughing, or seeding, enabling colonization of new surfaces and perpetuating infection cycles [8].

Antimicrobial resistance associated with biofilms poses a significant clinical challenge, particularly in infections caused by pathogens such as *Pseudomonas aeruginosa*, *Escherichia coli*, *Staphylococcus aureus*, *Klebsiella pneumoniae*, *Acinetobacter baumannii*, and *Staphylococcus epidermidis* [5]. A primary mechanism underlying this resistance is the limited penetration of antimicrobial agents, as the EPS matrix acts as a physical barrier, reducing drug access to embedded cells [9,10,11]. The glycocalyx can sequester up to 25% of administered antibiotics, further diminishing their efficacy [12]. In addition, the reduced metabolic activity of biofilm-associated cells decreases the effectiveness of antibiotics, which predominantly target actively proliferating bacteria [13]. Genetic adaptability within biofilms further exacerbates antimicrobial resistance. In *P. aeruginosa*, for instance, upregulation of the *ndvB* gene promotes the synthesis of glucans that sequester antibiotics from intracellular targets [14]. Biofilm development is also associated with increased production of heat shock proteins, enhancing bacterial survival under antibiotic stress [15]. Furthermore, intercellular communication and the secretion of extracellular vesicles contribute to collective antibiotic tolerance. Simultaneously, the dissemination of biofilm-derived cells, while enhancing motility, does not inherently reinstate antibiotic susceptibility, as these cells frequently preserve resistance characteristics acquired in the biofilm milieu [16]. Biofilm-associated bacteria also demonstrate resistance to components of the humoral immune system, complicating host clearance mechanisms [17].

In clinical practice, biofilm-associated infections significantly hinder antimicrobial therapy and elevate the risk of persistent or recurrent disease. Catheter-associated candidemia caused by high biofilm-forming *Candida* species is strongly linked to treatment failure, with persistent bloodstream infections showing an adjusted odds ratio of 8.0 despite antifungal therapy [18]. Similarly, *S. aureus* biofilms isolated from periprosthetic joint infections exhibit significantly elevated minimum inhibitory concentrations and minimum bactericidal concentrations, with only rifampin, doxycycline, and daptomycin demonstrating reliable bactericidal activity [19]. Within cystic fibrosis lungs, polymicrobial biofilms composed of *P. aeruginosa* and *Stenotrophomonas maltophilia* interact synergistically to enhance virulence and resistance, worsening pulmonary decline [20].

Epidemiological data show that biofilm-producing *Staphylococcus* strains, particularly those associated with invasive infections, display increased methicillin and fluoroquinolone resistance [21]. In chronic wounds, biofilm presence has been reported in 80% of bacterial isolates, with both multidrug-resistant and susceptible strains demonstrating comparable biofilm-forming abilities [22]. Notably, these biofilms sustain persistent polymicrobial colonization, underscoring their role as a major virulence determinant irrespective of antimicrobial resistance profiles [22]. These characteristics render biofilm-associated infections exceptionally difficult to treat, highlighting the urgent need for innovative therapeutic strategies targeting biofilm-specific mechanisms [23]. By enabling immune evasion, genetic diversification, and antibiotic resistance, biofilms establish protected infection niches that are exceptionally difficult to eradicate. These same structural and biochemical defenses also influence the formation and activity of neutrophil extracellular traps. Understanding how biofilm components, such as eDNA and microbial enzymes, interact with NETs is critical for deciphering the persistence of chronic infections. It is also important for designing therapies that target both microbial and immune contributions to biofilm stability.

## 3. NETosis: Mechanisms and Functions

### 3.1. Pathways of Netosis

Neutrophils, the most abundant myeloid leukocytes in circulation, serve as the first responders to sites of infection or tissue injury. Their antimicrobial functions rely on three major mechanisms: phagocytosis coupled with reactive oxygen species (ROS) generation, the release of cytotoxic granule contents such as neutrophil elastase (NE) and myeloperoxidase (MPO), and the formation of NETs [24,25]. First described by Brinkmann et al. in 2004 [26], NETs are web-like structures composed of decondensed chromatin fibers studded with antimicrobial granular proteins, histones, and cytoplasmic factors [27,28]. The process of NET formation termed as NETosis represents a distinct form of neutrophil activation and death, separate from apoptosis and necrosis.

**Figure 1:**
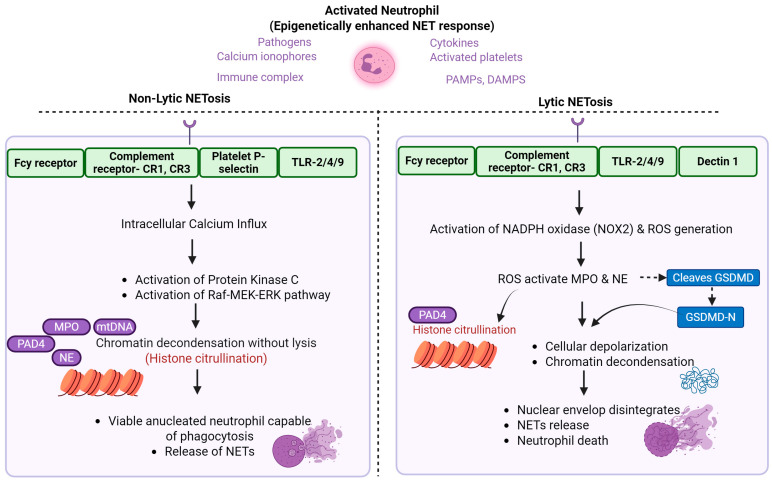
Mechanism of NETosis. NETosis occurs via two distinct pathways: non-lytic (vital) and lytic (suicidal). Various stimuli including pathogens, calcium ionophores, cytokines, activated platelets, PAMPs, DAMPs, and immune complexes can initiate NET formation. Trained neutrophil phenotypes, generated through prior inflammatory or microbial exposure, may exhibit heightened NETosis responses to these stimuli. These activating signals are recognized by receptors such as Fcγ receptors, complement receptors, platelet P-selectin, Toll-like receptors (TLRs), and Dectin-1, which collectively trigger an intracellular calcium influx. The rise in intracellular calcium activates peptidylarginine deiminase 4 (PAD4), leading to histone citrullination and chromatin decondensation. In non-lytic NETosis, the decondensed chromatin is packaged into vesicles and released without compromising the integrity of the plasma membrane, allowing the neutrophil to remain viable. Non-lytic NETosis may also involve rapid mitochondrial ROS production and the extrusion of mitochondrial DNA, generating mtDNA-rich NETs while preserving cell viability. In contrast, lytic NETosis involves a marked increase in reactive oxygen species (ROS) generation via activation of the NADPH oxidase (NOX) complex. Elevated ROS promote the release and activation of neutrophil elastase (NE) and myeloperoxidase (MPO) from cytoplasmic granules. NE-mediated cleavage of gasdermin-D (GSDMD) generates membrane pores that facilitate cellular swelling and are essential for terminal chromatin extrusion during lytic NETosis. Together with PAD4, NE and MPO facilitate histone H3 citrullination (CitH3) and further chromatin decondensation. Eventually, the nuclear and plasma membranes rupture, releasing NETs into the extracellular space, and the neutrophil undergoes cell death. Created in BioRender. Kumar, V. (2025) https://BioRender.com/esf9u15 (accessed on 30 November 2025).

NETosis occurs via two primary pathways: suicidal (lytic) and vital (non-lytic) NETosis (Figure 1). In lytic NETosis, extrinsic stimuli such as microbial infections, oxidative stress, or inflammatory mediators activate neutrophil surface receptors, including Toll-like receptors (TLRs), Fcγ receptors, and complement receptors. These signals initiate intracellular cascades (protein kinase C, Raf-MEK-ERK, and calcium influx), culminating in the formation of ROS mediated by NADPH oxidase (NOX2) [29]. ROS triggers disintegration of the nuclear envelope and the nuclear translocation of NE and MPO, which degrade histones and promote chromatin decondensation [24]. The decondensed chromatin, now decorated with NE, MPO, cathepsin G, and antimicrobial peptides, is released into the extracellular space following plasma membrane rupture. Simultaneously, the calcium-dependent enzyme peptidyl arginine deiminase 4 (PAD4) catalyzes the citrullination of histones H3 and H4, neutralizing their positive charge and weakening DNA-histone interactions, further facilitating chromatin relaxation [30].

Vital NETosis, in contrast, allows neutrophils to release NETs without undergoing lysis, preserving their phagocytic and secretory functions [31]. It can be triggered by bacterial components or activated platelets and may occur independently of ROS, involving vesicular transport of nuclear DNA. Stimuli such as monosodium urate crystals and bacterial lipopolysaccharide (LPS) can also induce NETosis via NADPH oxidase-independent pathways [32,33]. Although, many murine studies indicate that PAD4-mediated histone citrullination is required for NET formation [34,35]. However, human neutrophil studies demonstrate that certain physiological stimuli can induce NETosis that is ROS-independent [36] and in some instances even PAD4-independent or partially independent [37].

### 3.2. Stimuli and Signaling Pathways Regulating NETosis

Recent discoveries designate citrullinated histone H3 (CitH3) as a crucial effector and biomarker of NETosis, connecting PAD4-mediated histone modification to both acute NET formation and persistent immunological dysregulation [38]. Notably, PAD4-deficient mice do not always exhibit impaired bacterial clearance or worsened mortality, suggesting that PAD4’s necessity depends on infection type, stimulus, or timing [39]. These conflicting observations across species, stimuli, and in vitro vs. in vivo models argue strongly for the standardization of NETosis assays, including consistent stimuli, readouts (e.g., histone citrullination, DNA expulsion, ROS measurements), and cross-model comparisons.

Emerging evidence highlights the diversity of non-lytic NETosis pathways. Bacterial extracellular vesicles, such as outer membrane vesicles from oral pathogens, can trigger NETosis via endocytosis and cytosolic delivery of LPS, bypassing surface TLRs and instead activating the caspase-4/5/11–gasdermin D (GSDMD) axis [40]. This results in a more potent NET response than soluble LPS, which typically signals through the MEK/ERK cascade. Notably, neutrophils from patients with periodontitis exhibit enhanced NET release upon OMV exposure, likely due to epigenetically primed (trained immunity) mechanisms. In addition, DNA damage response pathways have also been implicated in NETosis [41].

### 3.3. Functional Roles of NETs in Host Defense and Inflammation

Functionally, NETs serve to trap and neutralize invading pathogens, limiting their dissemination and promoting microbial clearance. In vivo imaging studies show that NETs can physically confine bacterial biofilms and prevent deeper tissue invasion [4,42]. NET components such as IL-8 and TNF-α amplify local immune responses by recruiting and activating other leukocyte populations [43]. However, dysregulated NETosis is increasingly recognized as a driver of host tissue damage and chronic inflammation. In chronic infection conditions, neutrophil dysfunction frequently compromises the antimicrobial effectiveness of NETs. In cystic fibrosis (CF), neutrophils demonstrate compromised oxygen-dependent bactericidal mechanisms, notably diminished hypochlorous acid synthesis, which undermines intracellular eradication and facilitates the survival of biofilm-forming bacteria in the CF airway [44]. In chronic obstructive pulmonary disease (COPD), persistent NET production correlates with airway obstruction, increased neutrophilic inflammation, and diminished NET clearance, fostering an environment conducive to prolonged bacterial colonization [42].

Excessive NET release or defective clearance also contributes to autoimmune pathogenesis. In systemic lupus erythematosus and rheumatoid arthritis, excessive or defective clearance of NETs exposes nuclear autoantigens, fueling autoantibody production and type I interferon responses [45]. Moreover, in COPD, cigarette smoke induces NET-derived DNA that activates TLR9 and cGAS pathways, sustaining NF-κB-driven inflammation in epithelial and dendritic cells [46]. Altogether, NETosis represents a highly regulated antimicrobial defense mechanism with dual roles in direct pathogen clearance and modulation of inflammatory responses. However, its dysregulation is associated with a spectrum of inflammatory and autoimmune diseases, underscoring the importance of precisely modulating NET formation for therapeutic benefit.

### 3.4. Host Regulation of NETosis

Host regulation of NETosis involves an integrated network of enzymatic clearance pathways, neutrophil heterogeneity, and systemic physiological modulators that collectively determine whether NETs function as antimicrobial defenses or become drivers of chronic inflammation. The efficacy of NET clearance is predominantly reliant on endogenous DNase I and DNase1L3, which degrade extracellular chromatin produced during NETosis, hence averting the accumulation of DNA-protein complexes in tissues [47]. Once NETs are fragmented by DNases, remnants are typically cleared by phagocytes such as macrophages or dendritic cells [47]. Components of NETs, including citrullinated histones, are efficiently recognized and degraded by phagocytic cells, and their persistence in tissues has been linked to impaired NET clearance in chronic infections [48]. In vitro studies demonstrate that monocyte-derived macrophages and dendritic cells effectively phagocytize NET fragments, a process frequently augmented by opsonization with complement component C1q [49]. The dysfunction of these clearance mechanisms leads to the buildup of eDNA and histones, which contributes to chronic inflammation, autoimmune, and vascular disease. Such impairments are extensively described in systemic lupus erythematosus, diabetes, and chronic pulmonary disease, where diminished DNase activity or impaired phagocytic clearance results in enduring NET structures that may integrate into microbial biofilms and sustain inflammatory signaling [47,50,51].

Neutrophil heterogeneity further influences NETosis outcomes; low-density neutrophils, common in chronic infections and autoimmune conditions, demonstrate enhanced spontaneous NET formation, while aged or CXCR4^++^ neutrophils possess an inherently elevated NETotic potential due to tissue retention and modified metabolic states [52,53]. Tissue-resident neutrophils, influenced by local cytokine gradients, hypoxic conditions, or metabolic signals, demonstrate unique NETosis thresholds that affect NET persistence in chronic infection microenvironments [54]. Systemic physiological variables also govern the formation and elimination of NETs. Hyperglycemia augments PAD4 activity, elevates histone citrullination, and simultaneously hinders DNase-mediated NET breakdown [55]. Age-related immunosenescence modifies neutrophil priming, extending NET persistence, whereas microbiome-derived metabolites such as bile acids and short-chain fatty acids influence neutrophil activation thresholds and inflammatory response [56]. Cortisol and estrogen, as hormonal factors, have context-dependent effects that can either enhance or decrease NETosis, contingent upon immunological and metabolic circumstances [57,58].

The deregulation of these host pathways has prompted the emergence of clinically relevant biomarkers that elucidate NET dynamics and differentiate protective from pathogenic NET responses. Circulating MPO-DNA complexes operate as a reliable surrogate for NET load and correlate with illness severity in chronic inflammatory and infectious diseases, indicating persistent neutrophil activation and compromised NET clearance [59]. CitH3, a defining result of PAD4-mediated chromatin remodeling, has surfaced as a sensitive marker of NETosis and a prospective therapeutic target in infection-related immune dysregulation, with increased levels documented in wound infections and other chronic infections [60,61,62]. Recent advancements in imaging techniques, such as confocal microscopy, intravital imaging, and probe-based fluorescence detection, facilitate the spatial observation of NETs inside biofilm habitats and infected tissues, thereby improving diagnostic accuracy and enabling real-time evaluation of NET-pathogen interactions [63]. Integrating these biomarkers into clinical and translational research is crucial for differentiating protective from pathogenic NET responses and may inform the development of precision treatments for persistent infections.

## 4. Interaction Between NETs and Biofilms

**Figure 2:**
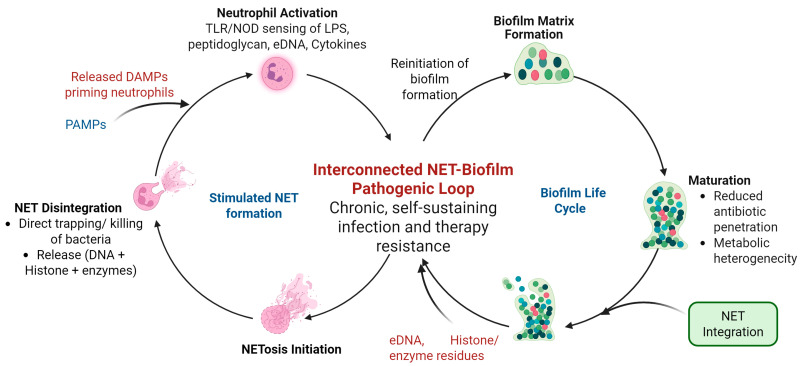
Dual self-reinforcing cycles of neutrophil NETosis and biofilm maturation in chronic infection. Two interrelated biological cycles function collaboratively to maintain chronic infection. The left cycle depicts the neutrophil response, wherein pathogen-derived signals (LPS, peptidoglycan, eDNA) activate neutrophils through TLR/NOD pathways. This activation initiates bactericidal mechanisms, including ROS production, degranulation, and phagocytosis, culminating in NETosis. The NET release exhibits antimicrobial activity via DNA-protein scaffolds that can capture and eliminate bacteria. The right cycle illustrates the reciprocal response of biofilms: the matrix formation facilitates biofilm maturation, decrease antibiotic penetration, and contribute to metabolic heterogeneity. Components derived from NETs, including eDNA, histones, and enzymes, are integrated into the biofilm matrix, enhancing its structural cohesion and resistance. Biofilms secrete nucleases and antioxidant enzymes that degrade NETs and reduce neutrophil antimicrobial activity. The bidirectional amplification occurring between these cycles establishes a persistent, self-reinforcing pathogenic loop that sustains inflammation, enhances biofilm architecture, and hinders both host and therapeutic clearance. Created in BioRender. Kumar, V. (2025) https://BioRender.com/esf9u15 (accessed on 30 November 2025).

In biofilm-associated infections, NETs often surround microbial communities, restricting pathogen spread and applying localized antimicrobial pressure. However, the dense EPS matrix of biofilms limits NET infiltration and the effectiveness of their antimicrobial components, allowing microbes within the biofilm core to evade immune clearance [64]. Paradoxically, NET-derived eDNA integrates into the biofilm matrix, enhancing its structural integrity and resilience. This interconnected and reciprocal relationship is illustrated in Figure 2.

Biofilm eDNA originates from both lysis-dependent and independent processes. Cell death mediated by quorum sensing (QS), fratricide, bacteriophage activity, and antibiotic-induced lysis release DNA from dead cells, while live bacteria actively secrete DNA via membrane vesicles, type IV secretion systems, or specialized channels [65,66,67,68]. Host-derived DNA, primarily from NETs or cells lysed by bacterial toxins, also contributes to biofilm formation by enhancing mechanical stability, microbial aggregation, and horizontal gene transfer including the spread of antibiotic resistance genes. For example, *S. aureus* incorporates NET-derived DNA to reinforce biofilm stability and hinder immune clearance [69]. Similarly, *P. aeruginosa* uses both microbial and host-derived eDNA to promote biofilm development, oxidative stress resistance, and antibiotic tolerance [70,71]. Fungal pathogens like *C. albicans* also integrate NET DNA into biofilms, boosting adhesion, biomass, and antifungal resistance [72]. Other species such as *Burkholderia pseudomallei* and *Streptococcus pneumoniae* utilize eDNA including NET components to initiate biofilm formation and strengthen structure, especially in inflammatory environments [73,74]. Although NET derived DNA is widely reported to enhance biofilm integrity and persistence, evidence suggests that NETs can also exert disruptive or inhibitory effects under certain conditions. Notably, metabolites such as bacterial lactate abundant within biofilm microenvironments can serve as signaling molecules that further stimulate mitochondrial ROS dependent NETosis, potentially amplifying inflammatory feedback within chronic infections [75]. In early-stage biofilms or environments with high extracellular DNase activity, NET associated proteases and ROS can damage microbial communities and impede matrix maturation [76]. Similarly, DNase-mediated degradation of NET DNA has been shown to weaken bacterial infection and increase bacterial susceptibility to host defenses and antimicrobials [77]. Recent evidence also indicates that metabolic enzyme deacetylation can intrinsically modulate NET formation. Deacetylation of IDH1 and MDH1 enhances NETosis via OPA1-mediated autophagy, an effect reversed by HDAC6 inhibition [78]. The balance between NET-mediated killing and structural reinforcement therefore appears to depend on factors such as local enzyme concentrations, bacterial species, and host immune status. Clarifying this duality is critical for developing therapeutic strategies that dismantle biofilms without impairing the beneficial antimicrobial functions of NETs.

To counteract NET-mediated antimicrobial effects, biofilm-forming microbes deploy various evasion strategies, predominantly producing nucleases that degrade the DNA backbone of NETs, thereby dismantling their structure. A summary of key bacterial strategies to evade or degrade NETs is provided in Table 1. *S. aureus* produces the nuclease Nuc, which breaks down NETs and facilitates immune evasion [69]. *Streptococcus mutans* expresses DeoC to degrade NETs, promoting biofilm dispersal and protecting against neutrophil killing [79]. Other pathogens using secreted or membrane-bound nucleases to evade NETs include *Aeromonas hydrophila* [80], *Leptospira* spp. [81], *Neisseria gonorrhoeae* [82], *Streptococcus agalactiae* [83], *Streptococcus pneumoniae* [84], *Streptococcus pyogenes* [85], *Streptococcus sanguinis* [86], *Streptococcus suis* [86], *Vibrio cholerae* [87], and *Yersinia enterocolitica*, which produces Ca^2+^/Mg^2+^-dependent NET-degrading nucleases similar to Gram-positive pathogens [88]. These nucleases are crucial for immune evasion, promoting biofilm dispersal and enabling chronic infection persistence.

The biofilm matrix itself acts as a physical barrier restricting NET penetration and sequestering antimicrobial factors at the periphery. Additionally, some pathogens manipulate host responses to inhibit NET formation or trigger release of structurally altered NETs with reduced antimicrobial potency. For instance, *P. aeruginosa* secretes virulence factors that suppress NETosis or induce dysfunctional NETs [100]. Alterations in microbial surface charge or expression of proteins that disrupt NET binding further impair NET-mediated killing [101,102].

## 5. Immune Checkpoint Molecules in NET–Biofilm Interactions

Recent advances in immunology reveal that immune checkpoint molecules, traditionally associated with adaptive immunity, also regulate innate immune responses, particularly neutrophil activation and NETosis. In chronic infections marked by persistent biofilms and elevated neutrophil activity, these checkpoints act as crucial mechanisms for immune evasion and inflammatory regulation, presenting promising therapeutic targets. Molecules such as PD-L1, CD47, and Siglecs are increasingly recognized for modulating neutrophil functions, with evidence that biofilms exploit these pathways to undermine host defenses.

The programmed death-1 (PD-1)/programmed death-ligand 1 (PD-L1) pathway, classically recognized for suppressing adaptive T-cell immunity, also exerts significant control over neutrophil activation and NETosis during chronic infections. PD-1, expressed on neutrophils, macrophages, and lymphocytes, binds to PD-L1 on immune or epithelial cells to deliver inhibitory signals that reduce oxidative burst, cytokine production, and NET release [103]. Its expression increases in neutrophils during chronic conditions like COPD and cystic fibrosis, where it suppresses inflammatory responses and supports infection persistence [104]. PD-L1 promotes NET formation by regulating autophagy through the PI3K/Akt/mTOR pathway, exacerbating tissue damage. In fungal infections such as *C. albicans*, β-glucans activate Dectin-1/JAK2/STAT3 signaling in neutrophils, inducing PD-L1 production [105]. This impairs neutrophil recruitment from bone marrow by enhancing autocrine chemokine secretion (CXCL1 and CXCL2), weakening antifungal defenses. Blocking PD-L1 or disrupting this signaling restores neutrophil release and improves infection outcomes, highlighting the context-dependent immunoregulatory role of neutrophil PD-L1. Experimental blockade of PD-1 or PD-L1 restores neutrophil recruitment, enhances NET clearance, and improves infection control in chronic sepsis and fungal infection models [106].

CD47 is a transmembrane glycoprotein delivering a “don’t eat me” signal via SIRPα on phagocytes, reducing phagocytosis and modulating innate immunity. In biofilm infections, bacteria like *S. aureus* induce CD47 expression on host immune cells, preventing phagocytic clearance and removal of NET debris. Neutrophils harboring live *S. aureus* maintain CD47 expression, evading macrophage efferocytosis despite apoptotic cues [107]. This prolongs neutrophil lifespan, impedes inflammation resolution, and supports biofilm persistence. *E. coli* K1 uses the CD47-TSP-1 axis to inhibit dendritic cell maturation, dampening immune responses and aiding bacterial survival in neonatal meningitis [108]. Therapeutically, blocking the CD47-SIRPα pathway restores phagocyte clearance of biofilm components and reduces inflammation, offering a viable adjunct for treating biofilm-related infections.

Siglec-9, a sialic acid-binding receptor on neutrophils, contributes to immune suppression during persistent infections. Many biofilm-forming pathogens produce sialylated glycoproteins that engage Siglec-9, inhibiting neutrophil activation, NET formation, and pro-inflammatory cytokine production [109]. Group B *Streptococcus* binds Siglec-9 in a sialic acid-dependent manner, suppressing neutrophil functions such as oxidative burst, phagocytosis, NET release, and bacterial killing [110]. In chronic lung infections, increased interaction between Siglec-9 and sialylated biofilm matrix components hinders neutrophil responses and promotes pathogen persistence [111]. Recent studies suggest biofilms can dynamically increase sialic acid production, amplify Siglec-9-mediated immunosuppression and gradually weaken host defenses [110].

Interactions between immune checkpoint molecules and neutrophils reshape the immune environment during chronic biofilm infections. Biofilms create an immunosuppressive niche by upregulating PD-L1, inhibiting NETosis, prolonging CD47-mediated neutrophil survival, and exploiting Siglec-9 to suppress inflammation. This network facilitates immune evasion, chronic inflammation, and tissue damage, thereby sustaining biofilm viability in host tissues.

## 6. Quorum Sensing Modulators Impacting NETosis

QS is a bacterial communication system that coordinates collective behaviors based on population density through signaling molecules called autoinducers. Beyond regulating biofilm formation and virulence, QS also modulates NET release during immune responses. In *P. aeruginosa*, mutations in the QS regulator *lasR* alter both the quantity and morphology of NETs produced, impacting host immunity [112,113,114]. QS-controlled proteases such as LasB and LasA further modulate inflammatory signaling and NET release [115,116]. QS molecules like 3O-C12-HSL and PQS inhibit immune cell growth including peripheral blood mononuclear and mast cells weakening host defenses [117,118].

Pathogens exploit QS beyond NETosis to impair immunity. For example, *Candida albicans* produces the QS molecule farnesol, which induces NET formation while also contributing to immune evasion [114]. Dysregulated NET production often driven by QS-regulated virulence factors can cause tissue damage and exacerbate inflammation in diseases like cystic fibrosis and sepsis [100,119,120]. QS molecules also influence neutrophil chemotaxis, oxidative burst, cytokine production, and modulate dendritic cell and macrophage activity [114,121]. This reflects a co-evolutionary arms race, where pathogens enhance QS dependent immune evasion while hosts adapt immune detection and response mechanisms [122]. These interactions shape infection outcomes and microbial community dynamics.

Given QS’s role in NET-related tissue damage, targeting QS pathways offers promising therapeutic potential. Anti-QS agents like furanones and phytochemicals have reduced bacterial virulence in pathogens such as *P. aeruginosa* and *Chromobacterium violaceum* in preclinical studies, without strong selective pressure for resistance [123,124]. QS inhibition disrupts virulence factor synthesis, impairs biofilm formation, and enhances pathogen susceptibility to immune clearance [125].

Advances in synthetic biology have led to synthetic QS inhibitors that block bacterial communication by antagonizing QS receptors or interfering with signal transmission, reducing virulence gene expression during critical infection stages [126,127,128]. Targeting QS represents a promising adjunctive strategy against infections caused by opportunistic pathogens. Importantly, QS affects both individual bacterial behavior and the dynamics of biofilm communities, thereby influencing their vulnerability to host immune defenses [129,130]. Understanding how QS regulates NETosis and immune modulation remains a rapidly advancing area with significant translational potential.

## 7. Pathogenic Synergy in Chronic Infections

One of the major contributors to the persistence of chronic infections despite host immune defenses and antimicrobial therapy is the synergistic relationship between biofilm formation and dysregulated NETosis. This interplay creates a self-perpetuating cycle of inflammation, immune evasion, and tissue injury, transforming protective immune responses into drivers of chronic pathology. Across diverse clinical settings, this pathogenic alliance underlies the persistence and recalcitrance of many infections.

Chronic lung infections, especially those induced by *P. aeruginosa*, are characteristic of cystic fibrosis, principally attributable to biofilm formation in the pulmonary airways. Studies demonstrate that biofilms in the lungs of cystic fibrosis patients exhibit resistance to host immune responses, facilitating persistent colonization by bacteria such as *P. aeruginosa*, which adapt to the inflammatory milieu created by neutrophil extracellular traps and other immune reactions [131,132,133]. The formation of these biofilms results in persistent inflammation and ultimately exacerbates respiratory deterioration as the immune system persistently responds inadequately to this biofilm-associated chronic infection [134]. In cystic fibrosis patients, the existence of biofilm not only facilitates the persistent survival of infections but also sustains the inflammatory cycle, leading to progressive lung tissue destruction.

In chronic wounds, biofilms created by various bacterial communities constitute a significant impediment to healing. About 80% of chronic wounds are inhabited by biofilm-forming bacteria, which induce sustained inflammation by perpetually attracting immune cells [135]. Opportunistic bacteria like *S. aureus* and *P. aeruginosa* utilize NET-derived eDNA to strengthen their biofilm architecture and improve resistance to antimicrobial drugs [69,79,136]. The continuous release of NETs fosters chronic inflammation, tissue injury, and a self-reinforcing inflammatory milieu that hinders wound closure and disrupts healing [72,79]. Interestingly, host metabolic regulators such as bile acids modulated by gut microbiota have been shown to influence neutrophil activation and immune homeostasis, suggesting a systemic axis that may impact NET-driven inflammation in chronic wounds [137]. Additionally, *P. aeruginosa* within biofilms synthesizes rhamnolipids upon interaction with neutrophils [138,139]. This in turn induce neutrophil necrosis, leading to the release of pro-inflammatory compounds that enhance neutrophil recruitment, as well as DNA and actin utilized for biofilm formation.

Periodontitis illustrates how polymicrobial biofilms exploit NETs to promote chronic oral inflammation. Pathogens within these biofilms destroy NET-associated antimicrobial proteins by secreted proteases while concurrently integrating NET-derived DNA into the biofilm structure, so augmenting its durability and promoting immune evasion [64,71]. NETs restrict oral inflammation by clearing both pathogen-associated molecular patterns (PAMPs) and damage-associated molecular patterns (DAMPs). During progression from periodontal inflammation to periodontitis, oral polymorphonuclear neutrophils (oPMNs) undergo marked alterations. Stimulated oPMNs show enhanced adhesion, microbial uptake, and a 13-fold increase in NET formation compared with circulatory PMNs [140]. They also display elevated influx, apoptosis, persistent hyperactivity, and reduced killing capacity [141]. NETs generated during periodontal infections significantly contribute to biofilm stability and resilience, establishing a cycle wherein inflammation promotes biofilm production, which then induces more inflammation and tissue damage, ultimately resulting in periodontal disease [142,143,144]. Pathogenic bacteria, like *Porphyromonas gingivalis* and *Fusobacterium nucleatum*, strengthen this association by co-aggregating to create intricate biofilms that withstand host defenses [145,146].

Biofilm formation on medical implants represents another domain where NETosis contributes to persistent infection. Infections associated with implanted medical devices are notoriously challenging to treat mostly because to the biofilms that develop on these artificial surfaces [147]. Bacteria within these biofilms can circumvent the immune response, and the ongoing creation of NETs due to chronic inflammation enhances biofilm stability and antibiotic resistance [148]. This situation is exacerbated by the immune system’s inability to adequately eliminate these chronic infections, which is influenced by ongoing inflammatory feedback loops caused by both the device and the related biofilm [147]. The interaction between NET production and biofilm persistence in chronic infections creates a detrimental cycle of inflammation that promotes pathogen survival and complicates treatment.

While treatment approaches aimed at NET–biofilm interactions present considerable potential, they also entail significant hazards that must be recognized. DNase-based methods can diminish NET-derived eDNA within biofilms but may unintentionally compromise microbial containment, as NET destruction has been demonstrated to impede bacterial clearance in specific infection models [149,150]. Similarly, therapies that facilitate biofilm dispersal without concurrent eradication may liberate planktonic bacteria with increased virulence potential, hence exacerbating infection severity. Immunomodulatory approaches, including PAD4 inhibition or checkpoint blockage, may impair critical innate immune activities, resulting in unexpected inflammatory outcomes or heightened vulnerability to infections [104,105]. These considerations highlight the need for therapeutic designs that balance biofilm disruption with preservation of critical host defense mechanisms.

## 8. Conclusions

The dynamic interplay between NETosis and biofilm formation represents a fundamental mechanism driving the persistence and pathology of chronic infections. While NETs serve as an essential first line of defense against invading pathogens, their components, particularly eDNA and histone-bound proteins, paradoxically reinforce biofilm structure, promote immune evasion, and sustain inflammation. This bidirectional relationship transforms an antimicrobial defense into a pathogenic mechanism that fosters microbial survival, tissue damage, and therapeutic resistance.

Recent advances have illuminated the complexity of this interaction. Biofilms, through QS molecules and metabolic cues, actively modulate neutrophil behavior, influencing the extent and quality of NET release. Conversely, dysregulated NETosis contributes to the formation of stable infection niches, as NET-derived DNA integrates into biofilm matrices. In parallel, immune checkpoint pathways such as PD-1/PD-L1, CD47-SIRPα, and Siglec-9 further regulate neutrophil activation and NET clearance, creating an immunosuppressive environment that favors chronic infection. Collectively, these interconnected systems highlight a multi-layered pathogenic network in which microbial signaling and host regulation converge to sustain infection.

Targeted approaches such as DNase therapy, PAD4 inhibition, and checkpoint blockade aim to restore immune balance and prevent excessive NET accumulation, while anti-QS agents disrupt microbial coordination and weaken biofilm integrity. However, these interventions also carry important risks. DNase-mediated NET degradation may impair microbial containment and facilitate pathogen spread; PAD4 inhibitors may blunt essential early innate responses; biofilm dispersal strategies risk releasing viable planktonic bacteria without ensuring clearance; and systemic immunomodulators such as checkpoint inhibitors can produce unintended inflammatory or immunosuppressive effects. These potential drawbacks highlight the need for careful therapeutic design and combined approaches that ensure both biofilm disruption and adequate immune protection.

Moving forward, research should prioritize integrative models that capture the spatial and temporal dynamics of NET–biofilm interactions, bridging molecular mechanisms with clinical manifestations. Identification of biomarkers distinguishing protective from pathogenic NET responses will be crucial for precision therapy. Ultimately, deciphering the NET–biofilm axis offers a path toward not only improved infection control but also a deeper understanding of how immune defense mechanisms can be co-opted into promoting chronic disease.

## Figures and Tables

**Table 1:** Mechanistic crosstalk Between NETosis and Biofilm Formation Across Major Pathogens.

Pathogen	NET Induction Mechanism	Biofilm Interaction with NETs	Immune Evasion Strategy	Associated Chronic Infections	References
*Pseudomonas aeruginosa*	Exopolysacch-arides (e.g., alginate), TLR activation	Uses NET DNA to reinforce EPS, increase resistance	DNases degrade NETs; rhamnolipids cause neutrophil lysis	Cystic fibrosis, chronic wounds, ventilator-associated pneumonia	[70,89,90]
*Staphylococcus aureus*	A-toxin, Protein A, Lipoteichoic acid activates NETosis	Incorporates NET-derived DNA into biofilm matrix	Secretes nuclease Nuc to dismantle NETs; Secretes leukocidins to cause lysis of neutrophils	Device related infections, osteomyelitis, diabetic foot ulcers	[69,91,92]
*Candida albicans*	Farsenol triggers NET formation	NET DNA enhances biofilm thickness, adhesion, antifungal resistance	Secreted aspartyl proteases (SAPs) and extracellular matrix inhibit NET activation	Catheter related candidemia, chronic mucocutaneous candidiasis	[93,94]
*Streptococcus pneumoniae*	Surface adhesins, pneumolysis	NET DNA stabilizes nasopharyngeal biofilms	Produces endonuclease EndA; degrades NETs	Otitis media, chronic sinusitis, pneumococcal pneumonia	[84]
*Burkholderia pseudomallei*	Inflammation-dependent NET induction	Integrates host eDNA into forming biofilms	Uses a Type 3 secretion system and capsular polysaccharide to inhibit NADPH oxidase and reduce NET release	Chronic pulmonary infections, melioidosis	[95]
*Escherichia coli*	TLR4 activation by LPS	NET DNA supports biofilm in urinary tract	Employs DanRI to inhibit neutrophil responses through the reduction in ROS production and NET formation	Chronic UTIs, catheter-associated infections	[96]
*Klebsiella pneumoniae*	LPS and capsular polysaccharides activate neutrophils	Biofilm entraps NETs at periphery	Encapsulated strains inhibit NET release	Lung abscess, chronic wounds	[97]
*Staphylococcus epidermidis*	Cell wall components stimulate NET release	Incorporates NET DNA into slime layer	Degrades NETs with protease	Prosthetic joint infections, catheter infections	[98]
*Acinetobacter baumannii*	Induces ROS-dependent NETs	NETs contribute to EPS in surface biofilms	Inhibits NET formation by restricting neutrophil adhesion	Burn wound infections, ventilator-associated pneumonia	[99]

## Data Availability

No new data were created or analyzed in this study.
